# Proximity-dependent initiation of hybridization chain reaction

**DOI:** 10.1038/ncomms8294

**Published:** 2015-06-12

**Authors:** Björn Koos, Gaëlle Cane, Karin Grannas, Liza Löf, Linda Arngården, Johan Heldin, Carl-Magnus Clausson, Axel Klaesson, M. Karoliina Hirvonen, Felipe M. S. de Oliveira, Vladimir O. Talibov, Nhan T. Pham, Manfred Auer, U. Helena Danielson, Johannes Haybaeck, Masood Kamali-Moghaddam, Ola Söderberg

**Affiliations:** 1 Uppsala University, Department of Immunology, Genetics and Pathology, Science for Life Laboratory, Biomedical center, Husargatan 3, Box 815, SE-75108 Uppsala, Sweden; 2 Department of Chemistry—BMC, Box 256, Uppsala University, SE-75123 Uppsala, Sweden; 3 School of Biological Sciences and School of Biomedical Sciences, University of Edinburgh, C H Waddington Building, Max Born Cresent, Kings Buildings, Edinburgh EH9 3BF, UK; 4 Institute of Pathology, Medical University of Graz, A-8036 Graz, Austria

## Abstract

Sensitive detection of protein interactions and post-translational modifications of native proteins is a challenge for research and diagnostic purposes. A method for this, which could be used in point-of-care devices and high-throughput screening, should be reliable, cost effective and robust. To achieve this, here we design a method (proxHCR) that combines the need for proximal binding with hybridization chain reaction (HCR) for signal amplification. When two oligonucleotide hairpins conjugated to antibodies bind in close proximity, they can be activated to reveal an initiator sequence. This starts a chain reaction of hybridization events between a pair of fluorophore-labelled oligonucleotide hairpins, generating a fluorescent product. In conclusion, we show the applicability of the proxHCR method for the detection of protein interactions and posttranslational modifications in microscopy and flow cytometry. As no enzymes are needed, proxHCR may be an inexpensive and robust alternative to proximity ligation assays.

## Abstract (web summary)

Proximity ligation assays are a sensitive method for detecting protein interactions, but require the addition of enzymes. Here the authors introduce proxHCR, an enzyme-free method of detecting interactions in close proximity by inducing a hybribization chain reaction (HCR) of fluorescently labelled oligonucleotides.

## Graphical abstract

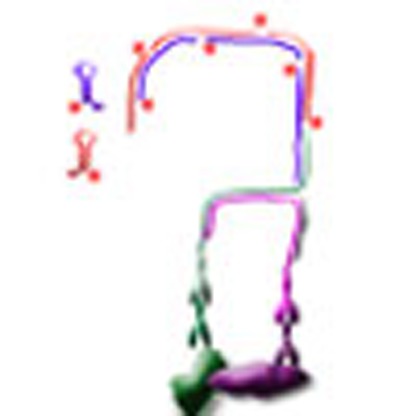

Ever since the sequencing of the human genome, cancer has been linked to genetic alterations. However, to understand cancer we need to unravel the molecular mechanisms these alterations code for. Transcriptome analysis will provide details on expression levels of genes, thereby providing information on the regulation of transcription and enable identification of different splice variants. This information will be propagated to the proteome where the levels of the different proteins will continue to be influenced by translation and degradation. As the activity of proteins in most cases is dependent on posttranslational modifications (PTMs) and protein–protein interactions (PPIs), analysis of these two events will provide information to determine the functional status of a cell or a signalling pathway. Hence, methods for selective and sensitive detection of proteins, PTMs and PPIs are highly warranted in clinical diagnostics, both in body fluids and in tissue sections. There are several methods available based on protein fragment complementation, for example, bimolecular fluorescence complementation, mammalian-membrane two-hybrid assay, mammalian protein protein interaction trap and kinase substrate sensor, which are very useful for discovering and analysing PPIs using ectopic expression systems^1^,^2^. For *in situ* analysis of endogenous proteins, targeted approaches using affinity reagents, for example, antibodies, are required. To increase selectivity of affinity reagent-based methods, multiple recognition events can be applied to overcome the problem with cross-reactivity. Detection of low abundant molecules will require either a sensitive instrument or powerful signal amplification. Proximity ligation assay (PLA) combines multiple recognition events with potent signal amplification. The method is based on pairs of proximity probes (that is, antibodies conjugated to strands of DNA) to detect the proteins of interest. Only on proximal binding of these probes can an amplifiable DNA strand be generated by ligation, which then is amplified by PCR^3^,^4^. For localized detection, rolling circle amplification (RCA), an isothermal DNA amplification technique, may be used^5^. RCA amplifies a circular template and generates long DNA strands that collapse into bundles of DNA. These bundles can be visualized by hybridizing fluorophore-labelled oligonucleotides to them, making it possible to detect single molecules *in situ*. Although PLA has a variety of advantages, one of the main disadvantages is the dependence on enzymes, which makes the method expensive and puts demands on storage and stability of enzymes. An approach to detect PTMs and protein interactions, without enzymatic steps, would be beneficial, especially for the development of point-of-care devices and high content screening.

An enzyme-free signal amplification method employing oligonucleotides has recently been published^6^. This method is called hybridization chain reaction (HCR) and is based on at least two different kinetically trapped hairpin structures. In absence of an initiator nucleic acid, both hairpin structures remain as monomers. However, once the initiator is introduced it will hybridize to one of the hairpin structures and invade it in a random walk process. This process is driven by the release of potential energy stored in the hairpin structure of the oligonucleotide^6^. The hybridization between the initiator and the first hairpin structure releases a part on the first hairpin structure that can bind and invade the second hairpin structure, while this can in turn bind and invade another molecule of the first species. The HCR will generate a long, nicked double-stranded DNA molecule, which will continue to grow linearly until the hairpin species are exhausted. In the initial paper, the technique could either be used to detect a target nucleic acid strand or to detect small biomolecules such as ATP by combining HCR with aptamer technology. Since then, the technique has been adapted to be used in combination with antibodies^7^, enzymatic-assisted readout^8^, multiplexing^9^ and real-time analysis^10^.

In this study we could show that proximity-dependent initiation of HCR (proxHCR) is a generally applicable enzyme-free method to detect proteins, PTMs and PPIs.

## Results

### Design and *in-silico* analysis of the oligonucleotide system

**Figure 1:**
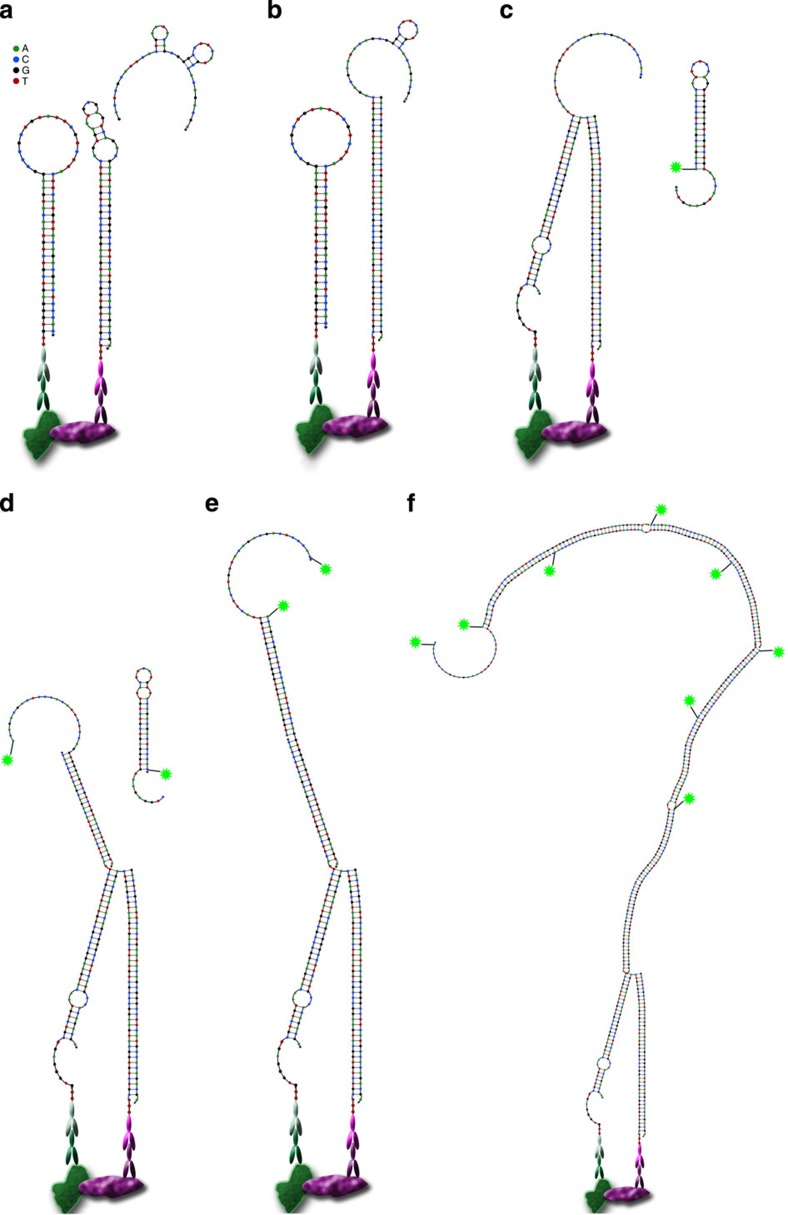
Principle of proxHCR. (**a**) Primary antibodies of two different species detect their respective targets and get bound by proximity probes (secondary antibodies conjugated to proximity hairpins). The activator is introduced. (**b**) Activator oligonucleotide binds in the loop of PH1 and invades the stem, thereby releasing the 3′-end of the oligonucleotide. (**c**) PH1 binds the loop of PH2 and invades it as well. The 3′-end of PH2 sticks out as a fishing rod, which serves as an initiator for the hybridization chain reaction. (**d**) One fluorescently labelled HCR hairpin (H1) molecule gets bound and invaded by this initiator and in turn binds and invades one molecule of H2 (**e**). (**f**) The HCR continues until there are no more HCR hairpin molecules left to hybridize.

To obtain a system where proximal binding of two probes would start an HCR, we had to design a two-step reaction. The first step would require proximal binding to make an oligonucleotide accessible that can in the second step facilitate the signal amplification brought about by HCR. In contrast to regular HCR^6^, this will require four hairpin species and an activator instead of just two hairpin species (Table 1). Activation of proximity sensing is brought about by the addition of an activator oligonucleotide that will invade the first proximity hairpin (PH1). This will liberate a bridging sequence in PH1 that will invade the second proximity hairpin (PH2) only if this is in close proximity. The bridged PH1–PH2 will exhibit the initiator sequence for HCR amplification, which previously was hidden in the stem of PH2 (Fig. 1).

**Figure 2:**
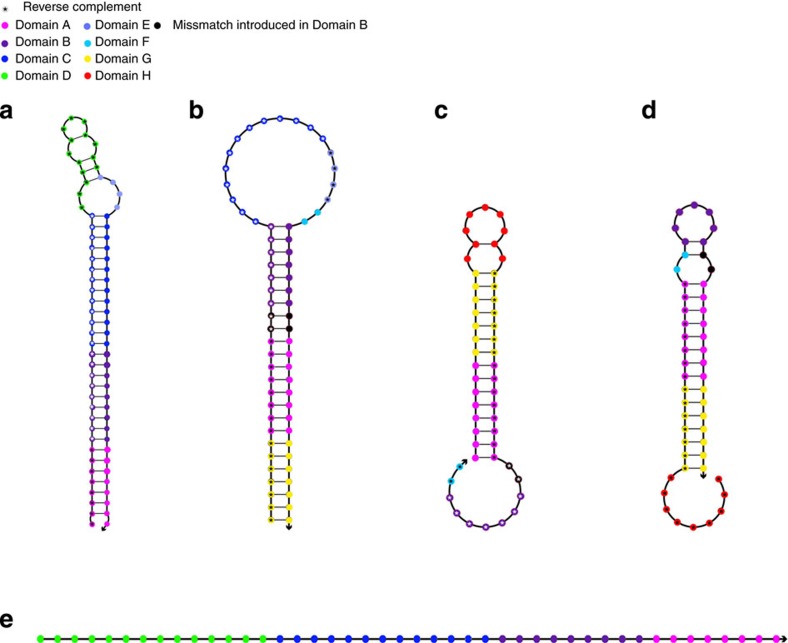
The different hairpin species. Proximity hairpin 1 (**a**) and proximity hairpin 2 (**b**) can be conjugated to the affinity reagents, to yield proximity probes. H1 (**c**) and H2 (**d**) are used for the amplification reagent and are labelled by a fluorophore or some other detection reagent. The reaction is started by the activator (**e**), which binds to PH1 and unlocks the bridging strand to bind at PH2. The different domains are colour coded to emphasize identical and reverse complementary regions in the different oligonucleotides.

Multiple optimization steps brought forward the sequences provided in Table 1. The optimization process is shown in Supplementary Table 1. The two arms adapt secondary structures with long stems (30 and 24 bp for PH1 and PH2, respectively) and relatively big loops (18 and 19 nt for the two proximity hairpins, respectively; Fig. 2a,b). The negative Gibbs free energy (–ΔG) for both secondary structures is estimated to be quite high (40 and 27 kcal mol^−1^ at 37 °C in 1 M NaCl). The HCR hairpins have similar structures to commonly used HCR oligonucleotides^6^. Namely a short sticky end (9 nt 5′ for H1 and 11 nt 3′ for H2), the stem is 15 bp for both oligonucleotides and the loops consist of 11 nt for H1 and 9 nt for H2 (Fig.2c,d). The two mismatches between the two proximity hairpins (position 16 and 17 of PH2) are worth mentioning. This mismatch is introduced to suppress generation of false-positive signal.

### Optimization of experimental parameters

**Figure 3:**
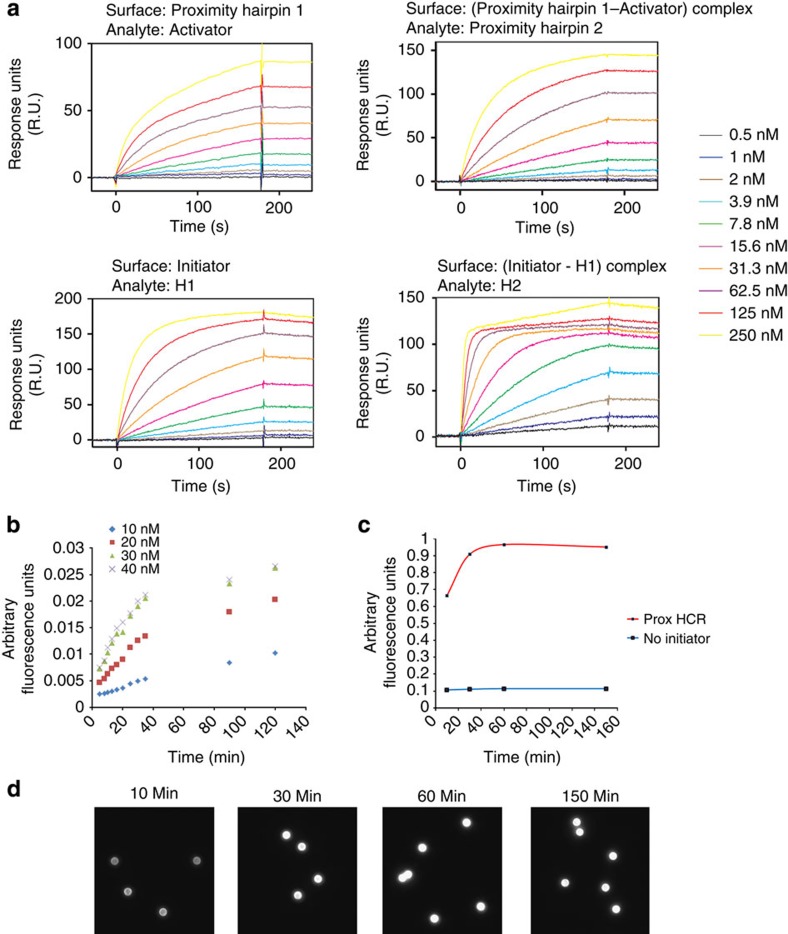
Kinetics of the oligonucleotide system. (**a**) The entire proxHCR process were broken down in four processes and evaluated using a SPR biosensor technology. Upper left corner: hybridization and invasion of the activator oligonucleotide to PH1 is reasonably fast and dissociation is not observed. Binding of this oligonucleotide complex to PH2 also shows high association and almost no reverse reaction (upper right corner). Binding of H1 to the initiator sequence (lower left corner) as well as binding of H2 to H1-initiator oligonucleotide complex is also strong (lower right corner). (**b**) In the Opera High Content Screening System, we further evaluated the properties of the used oligonucleotide system. Here, even after 5 min we could distinguish between 10 and 20 nM HCR oligonucleotides. The reaction slowed down after about 30 min with fluorescence only increasing marginally up to 120 min. These results could be confirmed using an epifluorescence microscope (**c**). After 10 min of amplification, a strong difference could be observed between positive and negative control. The difference continued to increase until 30 min, after which it stagnated, indicating depletion of HCR oligonucleotides. (**d**) Pictures of the positive beads at 10, 30, 60 and 150 min.

The first round of experiments was performed using surface plasmon resonance biosensor technology (SPR), to evaluate the kinetics of the interacting oligonucleotides. The experiments show an efficient binding of the activator oligonucleotide to PH1 (Fig. 3a). Furthermore, the subsequent binding of the opened PH1 to the PH2 also occurs efficiently without any measurable dissociation. Owing to the very slow dissociation (*k*_d_<10^−4^ s^−1^), complex affinity or kinetic rate constants could not be quantified. Next, we switched to the initiator sequence (Table 1), which is identical to the part that sticks out of the activator–PH1–PH2 complex and acts as an initiating oligonucleotide for the HCR. The results show fast association of H1 with the initiator, while again almost no dissociation could be observed. The same holds true for the H1–H2 interaction. Again, for both reactions no quantitative data could be obtained. Control experiments show no visible association between PH2 alone and H1 or H2. In addition, the activator–PH1 complex alone does not interact with H1 or H2 (Supplementary Fig. 1).

The amplification reaction was also evaluated in solution using polyacrylamide gel electrophoresis in combination with silver staining. The oligonucleotide system is found to result in fast and signal generation in the presence of the initiator oligonucleotide, while the system remains metastable in its absence (Supplementary Fig. 2).

To further characterize the behaviour of the amplification reaction, we used an Opera High Content Screening System to determine the buildup of fluorescence on beads during short reaction times. Our data show a robust increase in fluorescence after only 30 min of incubation at 37 °C. Even as little as 5 min of incubation is enough to see a dose-dependent (concentration of HCR oligonucleotides) increase in fluorescence (Fig. 3b).

Based on these experiments and the qualitative kinetic analysis from the SPR, we decided to use a concentration of 50 nM for each of the HCR oligonucleotides for subsequent experiments on beads.

Using epifluorescence microscopes, we can show that the reaction of 50 nM HCR oligonucleotides reaches its maximum fluorescence after 30 min and does not increase with longer incubation time (Fig. 3b). The fluorescence emission intensity is already distinguishable from the negative control after the earliest time point (10 min). Furthermore, the negative control does not generate a visible amount of fluorescence intensity over the whole time period.

### *In situ* analysis of protein interactions using proxHCR

**Figure 4:**
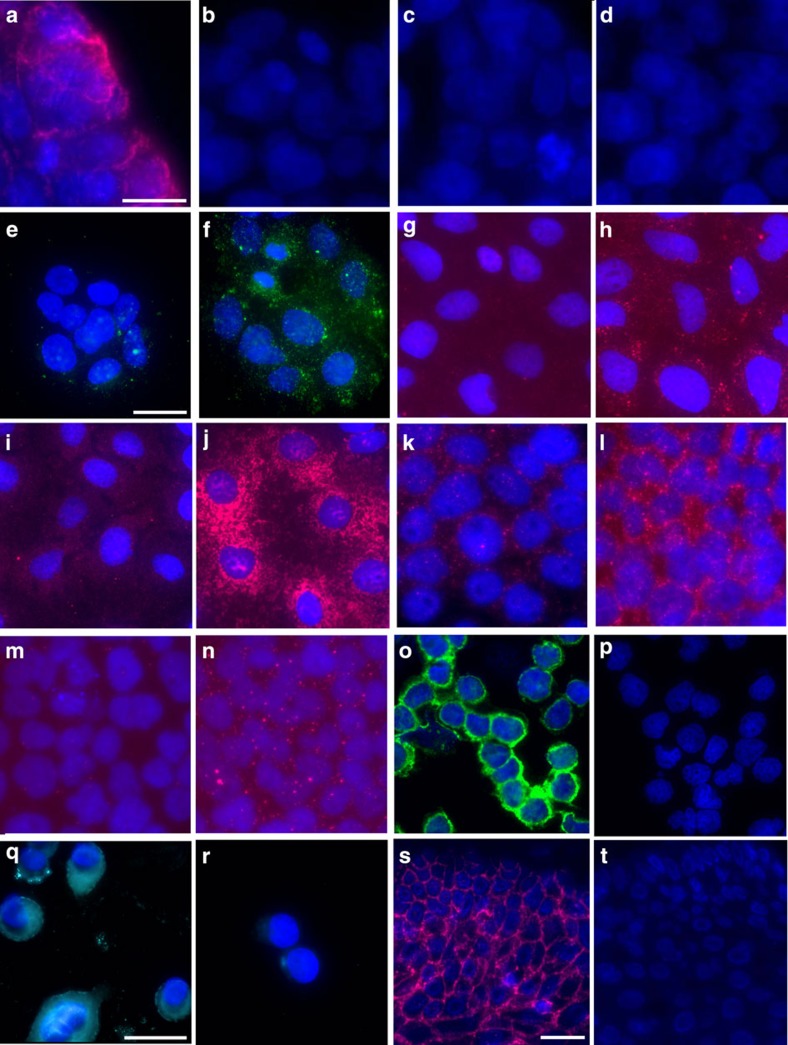
*In situ* prox-HCR. (**a–d**) Technical controls for the E-cadherin/β-catenin interaction. Strong membranous signal could be observed in HT29 cells when both primary antibodies were applied (**a**), while omitting either one of the primary antibodies (**b**,**c**) or both (**d**) did not yield visible signal. Phosphorylation of platelet-derived growth factor receptor-β (PDGFR-β) could be shown in BjHTert cells following stimulation with 100 ng ml^−1^ (**f**), while expression of phosphorylated receptor was low in non-stimulated cells (**e**). ProxHCR was used to visualize the induction of autophagy following starvation and incubation with CoCl_2_ in Caco cells (**g–j**). Although untreated cells showed only low basal activity (**g**) of BCL2-BNIP3 interaction, a highly increased signal could be observed in treated cells (**h**). The same holds true for LC3-SQSTM1 interaction (**i**,**j**). The MEK–ERK interaction could be induced in A431 cells by stimulation with 10 ng ml^−1^ EGF for 10 min (**l**), while the non-stimulated cells only showed low basal signal (**k**). Under the same conditions phosphorylation of Akt could be observed (**n**), while no phosphorylation was visible in non-stimulated cells (**m**). In panel **o**, the phosphorylation of Syk is shown (**p**: no primary antibodies). Detection of Her2 protein was possible as well and shown in panel **q** (**r**: no primary antibodies). Expression of E-cadherin/β-catenin interaction could be observed even in FFPE skin tissue (**s**), while no signal was generated when primary antibodies were omitted (**t**). White scale bars, 20 nm.

**Figure 5:**
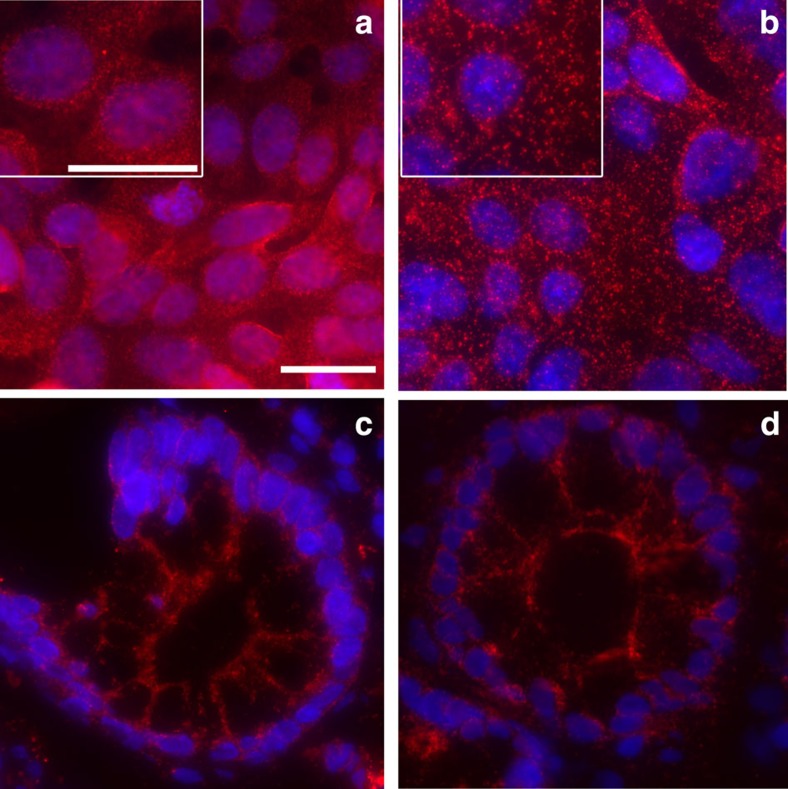
Comparison proxHCR with *in situ* PLA. Interaction between E-cadherin and β-catenin in DLD1 cells (**a**: proxHCR; **b**: *in situ* PLA). The inlay panels in the upper left corner of **a** and **b** show a 150% magnification for better evaluation of signal size and number. The same interaction in frozen colon tissue also resulted in comparable results between proxHCR (**c**) and *in situ* PLA (**d**). White scale bars, 20 nm.

To test the feasibility of proxHCR to record PPIs and PTMs *in situ*, we established a number of assays against known interactions and PTMs in a multitude of different cell lines. Figure 4 shows the results of these assays. The E-cadherin/β-catenin shows a strong membranous staining in HT29 cells when both primary antibodies are applied (Fig.4a), whereas omitting either one or both of the primary antibodies results in no detectable signal (Fig. 4b–d). We can further show that a variety of PPIs and PTMs can be visualized using proxHCR (Fig. 4e–o). Among them are membrane receptors such as phosphoplatelet-derived growth factor receptor-β (PDGFR-β) (Fig. 4e,f), indicators of autophagy (that is, BCL2/BNIP3 (Fig. 4g,h) and LC3/STQM3 (Fig. 4i,j)) and members of prominent receptor tyrosine kinase pathways (MEK/ERK interaction (Fig. 4k,l) and phosphorylation of Akt (Fig. 4m,n)). Phosphorylation of Syk in HG3 cells is also very nicely shown (Fig. 4o,p). The biological controls of the induced interactions still show low basal activity (Fig. 4e,g,i,k,m), whereas the technical controls (omission of primary antibody) do not show visible signal (Supplementary Fig. 3). Even single protein detection is possible using proxHCR (Fig. 4q,r). Here, Her2 is visualized using two primary antibodies and two proximity probes. Furthermore, we can show the feasibility of proxHCR for formalin-fixed paraffin-embedded (FFPE) skin tissue sections, staining for E-cadherin and β-catenin (Fig. 4s,t). We used the interaction between E-cadherin and β-catenin in DLD1 cells and in fresh-frozen colon tissue as a model system to compare proxHCR with *in situ* PLA (Fig. 5a–d). The results show the same specific pattern of signal localization for *in situ* PLA and proxHCR in cultured cells (Fig. 5 a,b) and in fresh-frozen colon tissue (Fig. 5c,d).

### Internalization of the EGF–EGFR complex in flow cytometry

**Figure 6:**
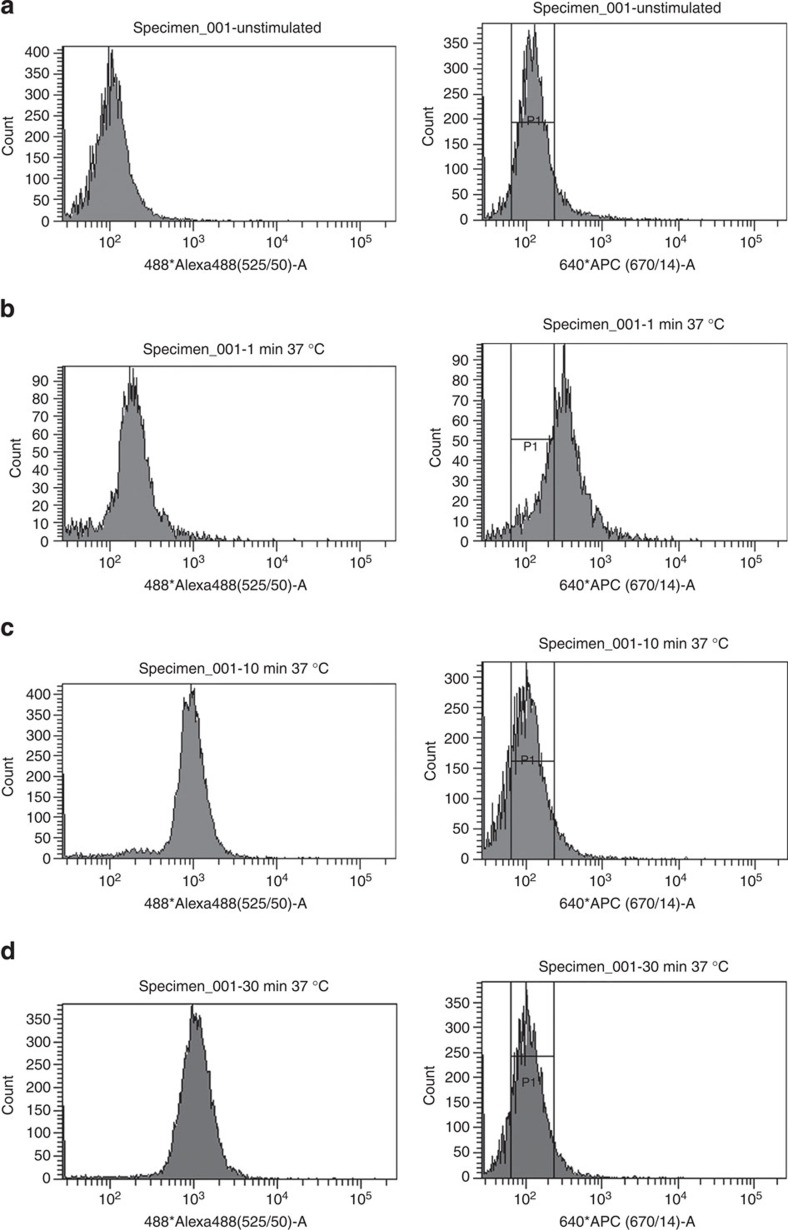
Flow-cytometric analysis of proxHCR on the model reaction of internalization of EGF/EGFR complex. Background levels of unstimulated cells (**a**) at 525 nm (AlexaFluor488) and 670 nm (Cy5). After 1 min of stimulation (**b**) with AlexaFluor488-EGF, a small shift at 525 nm can be observed. Furthermore, fluorescence at 670 nm shifts to higher values as well. After 10 min of stimulation (**c**), a large shift at 525 nm can be seen while fluorescence at 670 nm shifts back to background level. Prolonged incubation (30 min) did not alter the fluorescence levels significantly (**d**).

To determine whether proxHCR would also work with a flow cytometry readout, we set up an assay to detect the binding of epidermal growth factor (EGF) to EGF receptor (EGFR) in A431 cells (Fig. 6). The proxHCR assay is used to detect membranous AlexaFluor488-EGF/EGFR at 670 nm (Cy5), whereas internalized complexes would be detected by the AlexaFluor488 attached to EGF. Analysis of the flow cytometry data shows a small shift of fluorescence at 525 nm (AlexaFluor488) after stimulation with AlexaFluor488-EGF for 1 min at 37 °C (Fig. 6a,b). This shift increases after 10 min at 37 °C (Fig. 6c). In contrast, we can see an increase of the Cy5 fluorescence after 1 min at 37 °C (Fig. 6a,b), which decreases to baseline after 10 min of stimulation (Fig. 6c). Neither fluorescence at 525 nm nor at 670 nm change considerably after 30 min of incubation with AlexaFluor488-EGF (Fig. 6d).

## Discussion

In this study we present an enzyme-free method for the detection of PPIs and PTMs based on HCR. We could show its feasibility for *in situ* reactions on microscope slides and in flow cytometry as well.

Our proxHCR oligonucleotide system underwent several optimization steps to yield a system that shows a reasonably fast signal amplification rate without generating a false-positive signal. One of the features included is a two base mismatch between PH1 and PH2. This mismatch decreases the stability of the PH1–PH2 complex, which in turn makes the system less efficient. However, the mismatch is necessary to prevent the activator from binding to PH2 and to prevent the H1 and H2 from binding to the activator–PH1 complex, both of which might lead to unspecific signal.

One of the major differences of our method to classical HCR in the oligonucleotide system is the different stem/loop lengths. Dirks and Pierce^6^ propose in their initial paper an 18-bp stem and a 6-nt loop system, which differs from our system using 15 bp/9 nt and 15 bp/11 nt for the HCR hairpins and 24 bp/18 nt and 29 bp/18 nt for the proximity hairpins. When evaluating the stem-to-loop ratios, we can see considerable differences between the two systems (regular HCR ratio: 3 versus proxHCR ratio: 1.4 and 1.7). Dirks and Pierce^6^ tested a series of hairpins with different stem-to-loop ratios. All their hairpin variations were either unstable or too slow in starting the reaction. However, the system we present is stable for a long time and provides a suitable amplification rate. Interestingly, a recently published paper by Choi *et al*.^11^ uses different HCR amplifier systems that all build on a 12-nt loop with a 24- to 25-bp stem structure (stem-to-loop ratio: 2), which also performed well. Their estimated –ΔG values are comparable to those of our proximity hairpins (around 26–32 kcal mol^−1^ for theirs as compared with 40 and 27 kcal mol^−1^ for PH1 and PH2, respectively). This is considerably higher than the estimated –ΔG for the original hairpin structures by Dirks and Pierce^6^, which is around 21–22 kcal mol^−1^, which in turn is closer to our amplifier hairpins (18–19 kcal mol^−1^).

The amplification rate could however not be quantitatively determined. The reason for this is that the proposed mechanism for all interactions in the studied system includes a duplex invasion as an irreversible step. As the dissociation constant is <10^−4^ s^−1^, we were unable to determine either association or dissociation constant for which a detectable dissociation is necessary. Even though we cannot provide exact values, our in-solution experiments suggest that the HCR system amplifies rapidly, and that no visible dissociation occurs. This is also in accordance with the experiments we conducted on magnetic beads. Because of the lack of return reaction we can assume that the amplification continues until one or both of the HCR oligonucleotides are exhausted, which is a hallmark of HCRs^6^.

An HCR-based technique has recently been described in a proof-of-concept paper for detection of α-thrombin in solution^12^. In their approach the authors used a protector oligonucleotide (P) to block the HCR-initiating oligonucleotide (I2) in an aptamer against α-thrombin. If a second aptamer (I1) binds and, by conformational change, exposes a transducing DNA strand, the protector can be removed from the first aptamer, liberating the initiator oligonucleotide, and HCR can be initiated. This combination of using the conformational change of aptamers with introduction of limitations to the start of the HCR is elegant. However, the reliance on aptamers reduces the amount of possible applications, because aptamers are still less readily available compared with antibodies. An obstacle is that the I1 aptamer alone might bind to its protein and undergo conformational change. Once the transducing DNA sequence is accessible, it can displace any protector oligonucleotide available. This may take place even if the protector in question is in a P–I2 complex in solution. The then-freed I2 aptamer can start the amplification reaction without being bound in proximity of I1. This means that the assay might produce signal even if only the target for I1 is present in the sample. Hence, the sample will have to be incubated with P–I2 and excess unbound P–I2 needs to be removed before the addition of I1.

We have previously developed a method for visualization of protein interactions: *in situ* PLA^5^. In *in situ* PLA, two oligonucleotides are hybridized to two proximity probes and then ligated to form a circle. This circle is then amplified in a process called RCA to form a sub-micrometre-sized RCA product. The RCA products can be visualized by hybridizing fluorophore-coupled detection oligonucleotides. In this manner, *in situ* PLA generates bright signals that can be digitally quantified. Together with its high specificity and sensitivity, this is one of the main advantages of PLA (for review and variations of PLA, see Koos *et al*.^13^). However, as PLA relies on enzymatic steps (that is, ligation and enzymatic polymerization), the cost of enzymes is a hurdle, as is the need to store them at low temperature. Another drawback is the generation of non-circular ligation products, which will decrease the efficiency in molecular detections.

Both proxHCR and *in situ* PLA rely on the requirement of dual binding of proximity probes, to allow for the generation of a DNA molecule that can be amplified. We have herein shown that both these assays perform similarly. Furthermore, we could show that proxHCR provides selective assays for visualization of protein interactions and PTMs. When it comes to signal strength of the individual signals, it becomes clear that the enzyme-assisted amplification reaction of *in situ* PLA generates bigger and brighter signals, whereas proxHCR only creates very small dots of fluorescence. This is an advantage of the *in situ* PLA method, because it allows for digital quantification of its results. However, signal produced in proxHCR resembles much more a uniform diffuse staining. Quantification would hence be based on integrated fluorescence intensity per cell instead of digital enumeration of objects.

In addition to application of proxHCR in cells and tissues fixed on slides, we could show its feasibility in flow cytometry. We could distinguish between stimulated and unstimulated cells, and also visualize the binding of EGF to EGFR and its internalization into the cell. Alexa488-labelled EGF binds to its receptor on the plasma membrane where it is fixed and is accessible to the antibodies at early time points. Therefore, the proxHCR signal (Cy5) was higher than in unstimulated cells 1 min after stimulation. Furthermore, as the antibody against Alexa488 that we used might quench some of the fluorophore, the Alexa488 signal was not as strong after 1 min as compared with that after 10 min. As time progressed, the EGF/EGFR complex got internalized, which made it inaccessible for the antibodies (because the cells are not permeabilized). Therefore, the Cy5 signal was reduced at 10 min, compared with 1 min, after stimulation. Furthermore, the AlexaFluor488 that had been internalized was also inaccessible for the quenching antibody. Therefore, AlexaFluor488 signal rose after 10 min. The combination of high AlexaFluor488 signal and low Cy5 signal indicates that most of the EGF/EGFR complexes are internalized after 10 min. This time frame for the internalization of EGF/EGFR complexes is in accordance with results from other groups^14^.

The greatest advantage of the proxHCR method is its independence from enzymatic steps, while retaining the specificity of PLA. This reduces costs of the assay considerably, making the method better suited for high-throughput screening of protein interactions. Furthermore, it provides advantages by eliminating the control of enzyme quality after storage. We speculate that the largest impact of proxHCR might arise in point-of-care devices. A proxHCR device could be stored at room temperature (RT) for a long time and it could be run at 37 °C without the need of cycling temperatures. In addition, proxHCR is in theory amenable to other variations of HCR, such as the real-time HCR for quantification^10^ or the branched HCR amplification that might provide better signal to noise ratios due to its exponential signal growth^15^. Furthermore, the very nature of the hairpins, that is, the fact that they have secondary structures, makes them very sensitive to changes in oligonucleotide sequence. This has been nicely shown before^9^, suggesting that proxHCR (similarly to HCR) may be suitable to multiplex, which would be another advantage of the technique.

In conclusion, we have herein described a method that combines dual recognition, for increased selectivity or detection of PPIs and PTMs, with a non-enzymatic process of generating a localized signal.

## Methods

### Design of the oligonucleotide system

We first constructed all oligonucleotides *in silico* with NUPACK (www.nupack.org)^16^. We designed the oligonucleotide system in such a way that an activator oligonucleotide would invade the hairpin structure of the first proximity hairpin, which on proximity could invade the second proximity hairpin. Regarding both proximity hairpins, we designed them to be kinetically trapped in their hairpin structure in the absence of an activator oligonucleotide. The 3′-end of the second proximity hairpin in its opened form serves as an initiator and can invade the first of the two HCR-amplification oligonucleotides (HCR hairpins H1 and H2). By invading each other, H1 and H2 will build up a fluorophore-labelled detection molecule, in essence nicked and fluorescently labelled double-stranded DNA. Similar to the proximity hairpins, the HCR hairpins are designed to be trapped in their hairpin structure in the absence of the initiating sequence, thus avoiding self-initiation of the reaction (Fig. 1 and Table 1).

### Cell culture

We kept all cells in a humidified incubator at 37 °C, 5% CO_2_ atmosphere. We grew BjhTert cells (kind gift from Tarjei Mikkelsen) in Gibco minimum essential medium supplemented with 10% (v/v) fetal bovine serum, 2 mM L-glutamine, 100 U ml^−1^ penicillin–streptomycin. For slide preparation we trypsinized cells and seeded them into eight-well chamber slides (Lab-Tek, Nunc) before we allowed them to adhere for 48 h. At the second day the medium was exchanged for starvation medium (DMEM with 2 mM L-glutamine and 100 U ml^−1^ penicillin–streptomycin) and we incubated the cells for 24 h. For stimulation we added 100 ng ml^−1^ PDGF-BB to the cells and incubated for 5 min at 37 °C. Medium was then aspirated and cells were fixed in 3.7% formaldehyde solution for 30 min on ice. After washing away residual formaldehyde, we dried the slides in 96% ethanol and stored them at –20 °C until further use.

We maintained the DLD1 and HT29 cells (kind gifts from Tobias Sjöblom) in McCoy's 5A (Modified) medium (Gibco) supplemented with 10% (v/v) fetal bovine serum, 2 mM L-glutamine, 100 U ml^−1^ penicillin–streptomycin. For visualization of E-cadherin/β-catenin interaction we seeded the cells into eight-well chamber slides to reach near confluency after adherence. We incubated the cells for 24 h before aspirating the medium, washing with PBS and fixing for 30 min on ice with 3.7% formaldehyde solution. As described above, we washed cells, dried and stored them at –20 °C until further use.

HG3 cells^17^ (kind gift from Lary Mansouri) were kept in RMPI 1640 medium supplemented with 10% (v/v) fetal bovine serum, 2 mM L-glutamine, 100 U ml^−1^ penicillin–streptomycin. To prepare the slides cells were spun down (280*g* for 10 min) and washed with PBS. Cells (150,000) were transferred into each cell funnel in PBS and cytospins were created using the Cellspin 1 from Tharmac. Cells were fixed with 3.7% paraformaldehyde solution, washed and dried as described above. They were stored at –20 °C until further use.

We grew A431 cells (kind gift from Ingvar Ferby) in DMEM supplemented with 10% (v/v) FBS, 2 mM L-glutamine, 100 U ml^−1^ penicillin–streptomycin. For flow cytometry experiments we seeded them into six-well plates and left them to adhere overnight. The next day we starved the cells in starvation medium as described for BjhTert cells. For flow cytometry analysis we detached the cells using accutase (Sigma) at 37 °C for 10 min. We then washed the cells twice with PBSB (1 × PBS supplemented with 0.5% BSA) and stimulated them with 40 ng ml^−1^ AlexaFluor488-EGF for 1,10 and 30 min at 37 °C. The reaction was stopped by adding 1% formaldehyde solution. We then washed and properly fixed the cells with 1% formaldehyde solution for 10 min on ice. Residual formaldehyde was washed away after which cells were immediately used for proxHCR. For experiments on slides, we seeded A431 cells into eight-well chamber slides as described above. Cells were left to adhere before medium was removed and the cells were fixed with 3.7% formaldehyde solution as described above.

Caco-2 cells (kind gift from Tobias Sjöblom) were maintained in Gibco minimum essential medium supplemented with 20% (v/v) fetal bovine serum, 2 mM L-glutamine, 100 U ml^−1^ penicillin–streptomycin. Cells were seeded at 10^4^ cells per cm^2^ on chamber slides system as described above. When specified, cells were treated 24 h with 150 μM of Cobalt(II) chloride hexahydrate (Sigma-Aldrich) dissolved in PBS in the appropriate media without serum, to induce stress-dependent autophagy. One hour before the end of the treatment cells were treated with 100 nM of bafilomycin, to accumulate the autophagic vesicles by blocking their degradation. Cells were fixed in 3.7% formaldehyde solution as described above.

### Conjugation of antibodies—generation of proximity probes

We performed the conjugation of the secondary antibodies as described before^18^. Briefly, we activated anti-rabbit and anti-mouse antibodies (catalogue number: 711-005-152 and 715-005-150, respectively, Jackson ImmunoResearch Laboratories, West Grove, PA) with SANH (VWR) for 2 h at RT. We then removed the SANH using Zeba desalting column (Thermo Scientific) and mixed each batch separately with one of the aldehyde-modified proximity hairpins in threefold molecular excess. Before adding the proximity hairpins they were heated up to 95 °C for 2 min, to destroy any quaternary structures that may have formed. Using 10 mM aniline as a catalyst, we left the reaction at RT for an additional 2 h, before purifying the generated proximity probes by HPLC. The purity of all conjugates was verified by SDS gel electrophoresis (Supplementary Fig. 4).

### Hybridization interaction analysis using surface plasmon resonance biosensors

We performed all experiments using Biacore 2000 or Biacore S51 instruments. All buffers used in experiments were 0.22 μm filtered and degassed. To start with, we immobilized streptavidin on CM5 sensor chips via amine coupling chemistry as previously described, aiming at an immobilization level of 1,000–1,500 response units. One flow cell on each sensor chip was activated and deactivated without protein immobilization as a reference. We then preconditioned the streptavidin-coated surface with pulse injections of 50 mM NaOH, 1 M NaCl, to establish a stable baseline. To analyse the different interactions, we captured 150–200 or 50–100 RU of biotinylated oligonucleotides (initiator, PH1 or PH2) onto the streptavidin-coated surface. We performed all kinetic assays at 37 °C in HCR buffer (50 mM Na_2_HPO_4_, 1 M NaCl, pH 7.4) supplemented with 0.05% (v/v) Tween-20. After immobilization, a concentration series of 0.5–250 nM of the corresponding oligonucleotides (activator, PH2, H1 or H2) were injected over the surface with a flow rate of 45 μl min^−1^. For ternary complex studies (PH2–PH1–A and H2–H1–Initiator), we captured the ligand before the sample injection. All associations were monitored for 3 min, while dissociations were evaluated for 1 min. At the end of each cycle we regenerated the surface with a 30-s pulse injection of 10 mM NaOH. Surface decay was <1% per cycle. The sensorgrams for all experiments were double referenced using Biaevaluation v. 3.0 software (GE Healthcare, UK).

### Imaging of fluorescence buildup on magnetic beads

We loaded streptavidin-coated magnetic beads (Dynabeads M-280 Streptavidin, Invitrogen) with 2 nM biotinylated initiator in HCR buffer (50 mM Na_2_HPO_4_, 1 M NaCl, pH 7.4) and incubated them for 30 min at RT. In the meantime, H1 and H2 were thawed and separately left to adjust to RT for 30 min. We washed the beads twice with HCR buffer and then incubated them with increasing concentrations of H1 and H2 at 37 °C in 50 μl HCR buffer. Beads were transferred to a 384-well plate (Molecular Machines and Industries AG, MMI, Switzerland) and imaged in the Opera High Content Screening System (Perkin Elmer). We took 16 images of each condition using a 488-nm solid-state laser at 2 mW, to excite the samples through a × 60 UPLSAPO water-immersion objective with numerical aperture 1.2. The exposure time of the images was 160 ms and exposure height was set to 1.5 μm above the bottom of the well. Images were captured using a cooled CCD (charge-coupled device) camera with a 520 (±17.5)-nm bandpass filter.

### Validation of Opera High Content Screening System by epifluorescence microscopy

Streptavidin-coated magnetic beads were loaded with 10 nM biotinylated PH1 and PH2 as described above. After washing away the residual unbound hairpins, we split the reaction and incubated one half with 10 nM activator oligonucleotide for 30 min at 37 °C, while we left the other half in the reaction buffer. After two additional washing steps, we incubated both samples with 50 nM of amplification oligonucleotides. After predefined periods of time (10, 30, 60 and 150 min) samples were taken, washed, immobilized on a poly L-lysine-coated glass slide (Sigma) and visualized under the microscope (Axioplan, Zeiss).

### In-solution experiments

The performance of the oligonucleotide system was evaluated in solution by mixing 1 μM of each amplification hairpin (H1 and H2) in HCR buffer. To start the reaction, 0.1 μM or 0.03 μM initiator oligonucleotide was added. The initiator was added at different time points to result in different amplification times (0, 15, 30 and 60 min). The products of proxHCR were separated with a GenePhor system electrophoresis unit with EPS 600 power supply (Pharmacia LKB Biothechnology) on 12.5% GeneGel Excel polyacrylamide gel (17-6000-14, GE Healthcare, UK) at 14 °C, 600 V, 25 mA and 15 W for 100 min. Subsequently, the gel was stained using Pierce silver staining for mass spectrometry (24600), according to manufacturer's recommendations. For growth evaluation, a DNA ladder was used (Fast Ruler, SM1103, Thermo Scientific).

### *In situ* proxHCR experiments in fixed cells

We rehydrated the BjhTert slides and SKBR3 slides in PBS, permeabilized them with 0.1% Triton X-100 for 5 min and washed in PBS again. The cells were blocked using DuoLink blocking reagent (Olink Bioscience) for 45 min at 37 °C; thereafter, we incubated the cells with primary antibodies against PDGF receptor (1:100, 3169 S, Cell Signaling) and total phospho-tyrosine (1:100, pY-100, 9411 S, Cell Signaling) overnight at 4 °C. On washing twice with PBS at RT for 2 min and once with HCR buffer for 2 min, we incubated the slides with 10 μg ml^−1^ proximity probes directed against rabbit IgG and mouse IgG for 1 h at 37 °C in HCR buffer. After two more washing steps with HCR buffer, we incubated 10 nM of activator oligonucleotide for 30 min. We then, after a quick wash, incubated the cells with 20 nM HCR hairpins for 1 h at 37 °C in HCR buffer. Subsequently, we washed the slides twice before we stained the nuclei with Hoechst and then mounted the slides with SlowFade and a coverslip.

SKBR3 cells were rehydrated and permeabilized as described above. On 30 min of blocking with DuoLink blocking reagent, cells were incubated with primary antibodies against Her2 (1:10,000, A048529, Dako, and 1:1,000, 05-1130, Merck Millipore) overnight at 4 °C. We washed the cells with PBS and continued the proxHCR assay as described for BjhTert cells. SKBR3 slides were a kind gift from Olink Bioscience.

Regarding the DLD1, A431 and HG3 cells, we rehydrated and permeabilized them as described above. We blocked nonspecific binding sites with DuoLink blocking reagent for 1 h at 37 °C and continued by incubating the cells with antibodies directed against E-cadherin (1:100, 610182, BD Biosciences) and β-catenin (1:300, Sc7199, Santa-Cruz) for DLD1 cells, Mek (1:50, mab2678, RnD) and Erk1/2 (1:250, 06-182, Millipore), as well as pS473-Akt (1:50, 4060, Cell Signaling) and Akt (1:100, 2920, Cell Signaling) for A431 cells and primary antibodies directed against total Syk (1:100, Sc1240, Santa-Cruz) and p-Syk (1:200, AP3271a, Abgent) for HG3 cells overnight at 4 °C. On washing for 5 min with TBS–0.05% Tween-20 (PBS for A431 cells), we blocked nonspecific binding sites again for 15 min (for A431 cells, no such blocking was needed) and incubated the slides with 10 μg ml^−1^ proximity probes (3 μg ml^−1^ for Mek/Erk interactions and phosphorylation of Akt) directed against rabbit and mouse IgG for 1 h at 37 °C. Finally, we performed the initiation and amplification of proxHCR as described above.

After rehydration, Caco-2 cells were permeabilized in 0.4% Triton X-100 in combination with 0.05% CAPSO for 15 min. After washing with TBS-0.05% Tween, we blocked nonspecific binding sites using Duolink blocking reagent for 60 min at 37 °C. On this, we incubated primary antibodies against Bcl2 (1:20, ab77567, Abcam) and Bnip3 (1:20,PA5-11402, Pierce Antibodies) or Lc3 (1:20, 0231-100/LC3-5F10, Nanotools) and Sqstm1 (1:20, sc-25575, Santa Cruz Biotechnology) overnight at 4 °C. HCR protocol was performed as described for DLD1 cells.

### *In situ* PLA experiments

For *in situ* PLA experiments, the DuoLink kit (Olink Bioscience) was used according to manufacturer's recommendations. Briefly, DLD1 cells were rehydrated and permeabilized as described for proxHCR. On blocking of nonspecific binding sites with DuoLink blocking reagent for 1 h at 37 °C, we incubated cells with primary antibodies directed against E-cadherin (1:100) and β-catenin (1:300) overnight at 4 °C. Cells were washed with DuoLink Wash buffer A two time for 5 min and incubated with proximity probes (PLUS and MINUS, Olink Bioscience) in antibody diluent (Olink Bioscience) for 1 h at 37 °C. After a new round of washing steps, ligation mix was applied to the cells and incubated for 30 min at 37 °C. Additional washing was followed by RCA and fluorescent detection of the rolling circle products. Cells were counterstained with Hoechst and mounted as described above.

### Formalin-fixed paraffin-embedded tissue

FFPE human skin tissue was used to show feasibility of proxHCR in FFPE tissue. On deparaffination, antigens were retrieved using pH 6 target retrieval solution (S1699, Dako) for 10 min at 125 °C. Subsequently, slides were blocked using Olink block, dipped in PBS and primary antibodies (E-cadherin, 1:100, 610182, BD Biosciences and β-catenin, 1:300, Sc7199, Santa-Cruz) were added and incubated overnight at 4 °C. We washed the slides 3 × in PBS and added proximity probes directed against mouse and rabbit IgG for 1 h at RT. After subsequent washing in PBS, 10 nM activator was added in HCR buffer and incubated for 30 min at 37 °C. After another washing step in HCR buffer amplification hairpins (H1 and H2, 20 nM each) were added and incubated on the slide for 90 min at 37 °C. Finally, slides were washed in HCR buffer for 10 min, counterstained with Hoechst and mounted. In the negative control, primary antibodies were omitted.

The samples of this anonymized skin tissue was a kind gift of Olink AB and has previously been approved by the local ethical standards committee (Uppsala 2005:347).

### Frozen tissue

We fixed fresh-frozen healthy colon tissue using 3.7% formaldehyde solution at RT for 15 min. Slides were washed with PBS twice and then blocked using Duolink blocking reagent for 60 min at 37 °C. Subsequent to blocking, we incubated the slides with primary antibodies against E-cadherin (1:100) and β-catenin (1:300) in PBS overnight at 4 °C. The next day the slides were washed with PBS 3 × 2 min and blocked again for 30 min. We then incubated the slides with proximity probes directed against mouse and rabbit IgG for 1 h at 37 °C in HCR buffer. After two more washes with HCR buffer, the slides were incubated with activator oligonucleotides for 30 min at 37 °C. Slides were washed again twice with HCR buffer and slides were incubated with 20 nM HCR hairpins in HCR buffer for 1 h at 37 °C. Slides were subsequently washed with HCR buffer and once with PBS before nuclei were counterstained with Hoechst. Slides were mounted with SlowFade.

The tissue samples and respective data from the Medical University of Graz were provided by the Biobank Graz with ethics approval of the project under the ethical commission number 23-015 ex 10/11, entitled ‘Molecular and cellular characterisation of colorectal cancer'.

For epifluorescence image acquisition of assays run on cells and tissues, we used a Zeiss Axioplan 2 imaging microscope equipped with filters optimized for Texas Red, AlexaFluor 488, Cy5 and 4,6-diamidino-2-phenylindole, a × 40 (1.3 numerical aperture) objective and a Zeiss AxioCam MRm camera. We kept the exposure times the same for all samples within each experiment. For the *in situ* experiments, we acquired 10 *z*-levels with 330 nm in between. For all data sets we chose the sites for acquisition randomly.

### Flow cytometry

Cells were blocked using blocking buffer (Olink Bioscience) for 45 min before they were incubated with primary antibodies directed against EGFR (1:500, M723901, Dako) and Alexa Fluor 488 (1:500, A-11094, Life Technologies) at 4 °C overnight. After washing, the proxHCR protocol was carried out as described above and fluorescence was read out using the LSR-II (BD Bioscience).

### Image analysis

To achieve a measurement of the fluorescence of the magnetic beads in the images, which translates to signal production via HCR, we used CellProfiler 2.1.0 (ref. 19). We identified fluorescent beads as primary objects. Next, we measured the median intensity value for all pixels of the identified objects, which are translated into fluorescence units (that is, a normalization of the intensity values recorded at 8 bit to a scale from 0 to 1.)

## Additional information

**How to cite this article**: Koos, B. *et al*. Proximity-dependent initiation of hybridization chain reaction. *Nat. Commun.* 6:7294 doi: 10.1038/ncomms8294 (2015).

## Supplementary Material

Supplementary Information Supplementary Figures 1-4, Supplementary Tables 1-4.

## Figures and Tables

**Table 1:** ProxHCR oligonucleotide system.

Activator	5′-GACTCGCATTCACTGAATACAGCGGGCCTTCATGCCACAGACGA-3′
Proximity-hairpin 1	5′-AAAAATCGTCTGTGGCATGAAGGCCCGCTGTATTCAGTGAATGCGAGTCAGACGAATACAGCGGGCCTTCATGCCACAGACGA-3′
Proximity-hairpin 2	5′-AAAAAGTGGGAGTCGTCTGTAACATGAAGGCCCGCTGTATTCGTCTTACTTCATGTTACAGACGACTCCCAC-3′
HCR hairpin 1	5′-Fluorophore-ACAGACGACTCCCACATTCTCCAGGTGGGAGTCGTCTGTAACATGAAGTA-3′
HCR hairpin 2	5′-CTGGAGAATGTGGGAGTCGTCTGTTACTTCATGTTACAGACGACTCCCAC-Fluorophore-3′
Initiator	5′-TACTTCATGTTACAGACGACTCCCAC-3′
