# Supplementary material for: Proximity-dependent initiation of hybridization chain reaction
Source: Nat Commun. 2015 Jun 12;6:7294. doi: 10.1038/ncomms8294 (PMC4490387; doi:10.1038/ncomms8294)
Supplement: Supplementary Information — Supplementary Figures 1-4, Supplementary Tables 1-4. [file ncomms8294-s1.pdf]

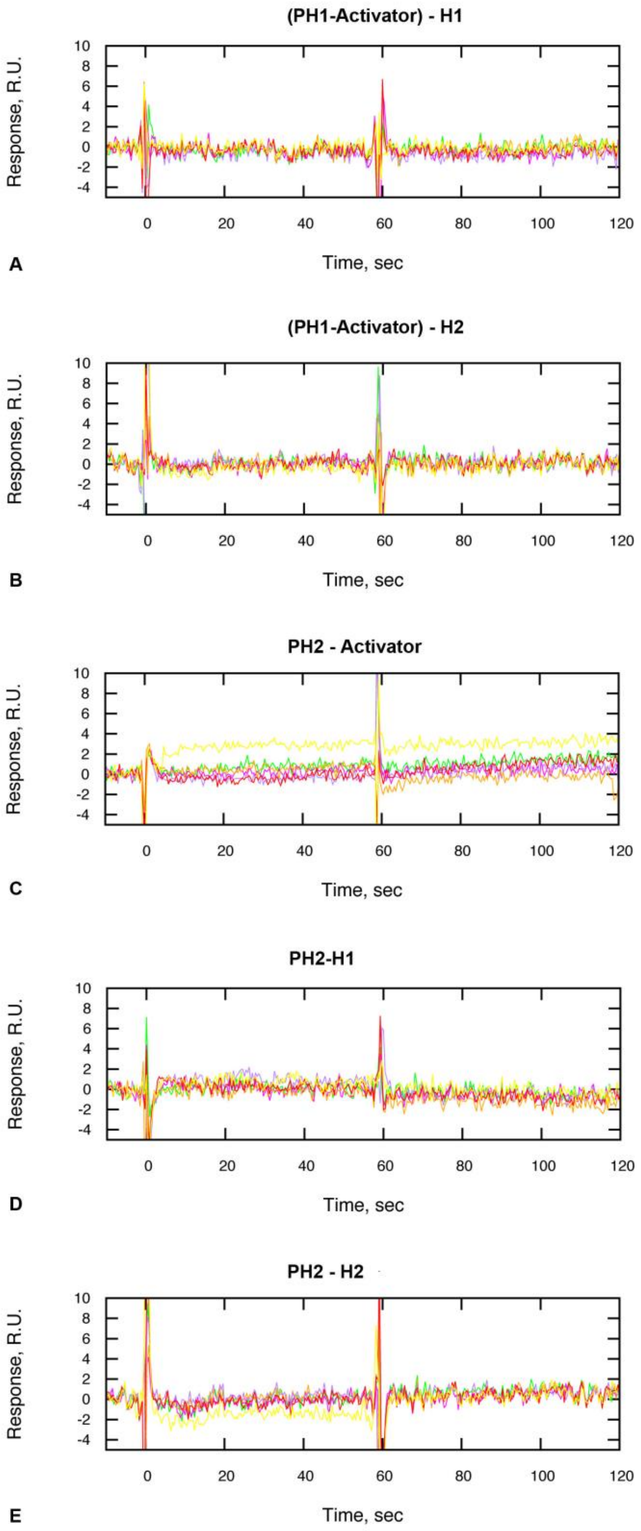

Supplementary Figure 1: Biacore negative controls. Unspecific interactions of the different oligonucleotides were evaluated with Biacore. We used a concentration range from 7.8 nM till 250 nM of all oligonucleotides. The PH1 – Activator complex was not sufficient to start the amplification process by either binding to H1 (**a**) or to H2 (**b**). Furthermore PH2 did not bind to the activator oligonucleotide (**c**) and not to H1 (**d**) or H2 (**e**). Therefore PH2 is incapable of generating signal by itself as well.

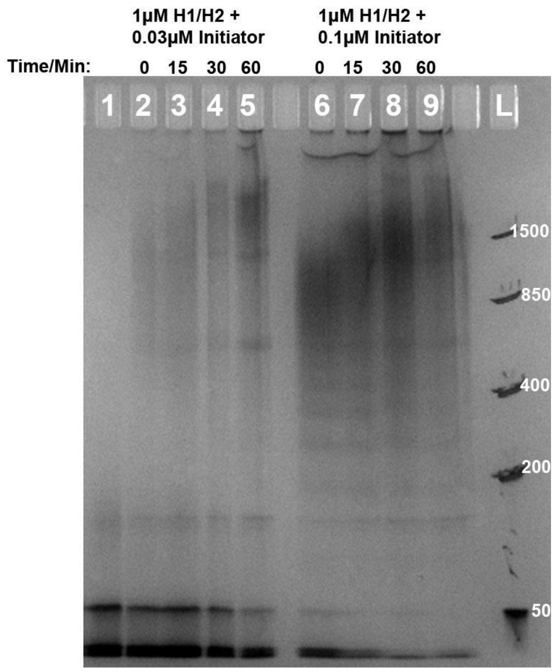

Supplementary Figure 2: Polyacrylamide gel electrophoresis to evaluate in solution performance of the oligonucleotide system. The HCR (1 μ M each) oligonucleotides are meta-stable in absence of the Initiator (**1**). After addition of 0.03 μ M Initiator to the mixture the HCR commences (**2-5**). The time points evaluated are 0 min (directly after adding the Initiator; **2**), 15 min (**3**), 30 min (**4**) and 60 min (**5**). Increasing Initiator concentration to 0.1 μ M increased reaction speed considerably (**6-9**). Already directly after addition of Initiator can high molecular weight products be observed (**6**) this product increases and growth after 15 min (**7**) to 30 min (**8**) and 60 min (**9**). The lane **L** depicts the ladder of dsDNA, ranging from 50 to 1500 bp.

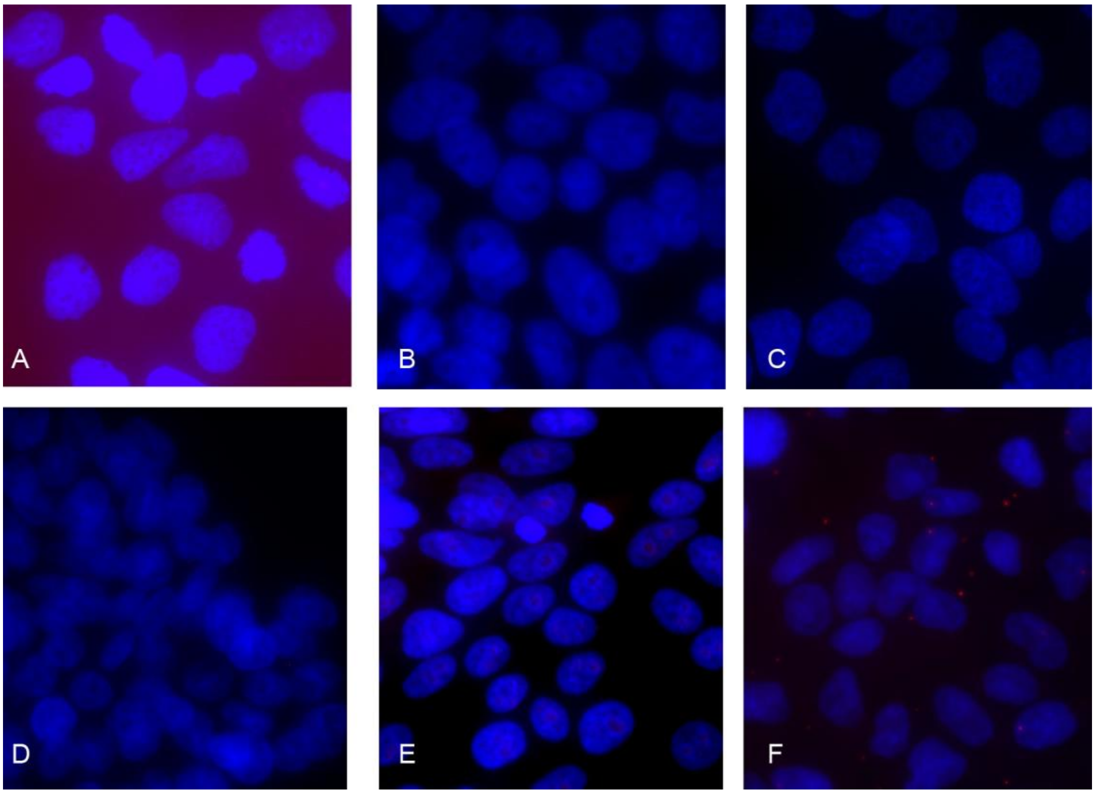

Supplementary Figure 3: Antibody negative controls. Omitting primary antibodies to test for unspecific binding of the proximity probes in Caco (**a**), A431 (**b**), BjhTert (**c**), HT29 (**d**), and DLD1 (**e**) resulted in no detectable proxHCR signal. The negative control for the PLA reaction in DLD1 cells (**f**) also resulted in minimal signal.

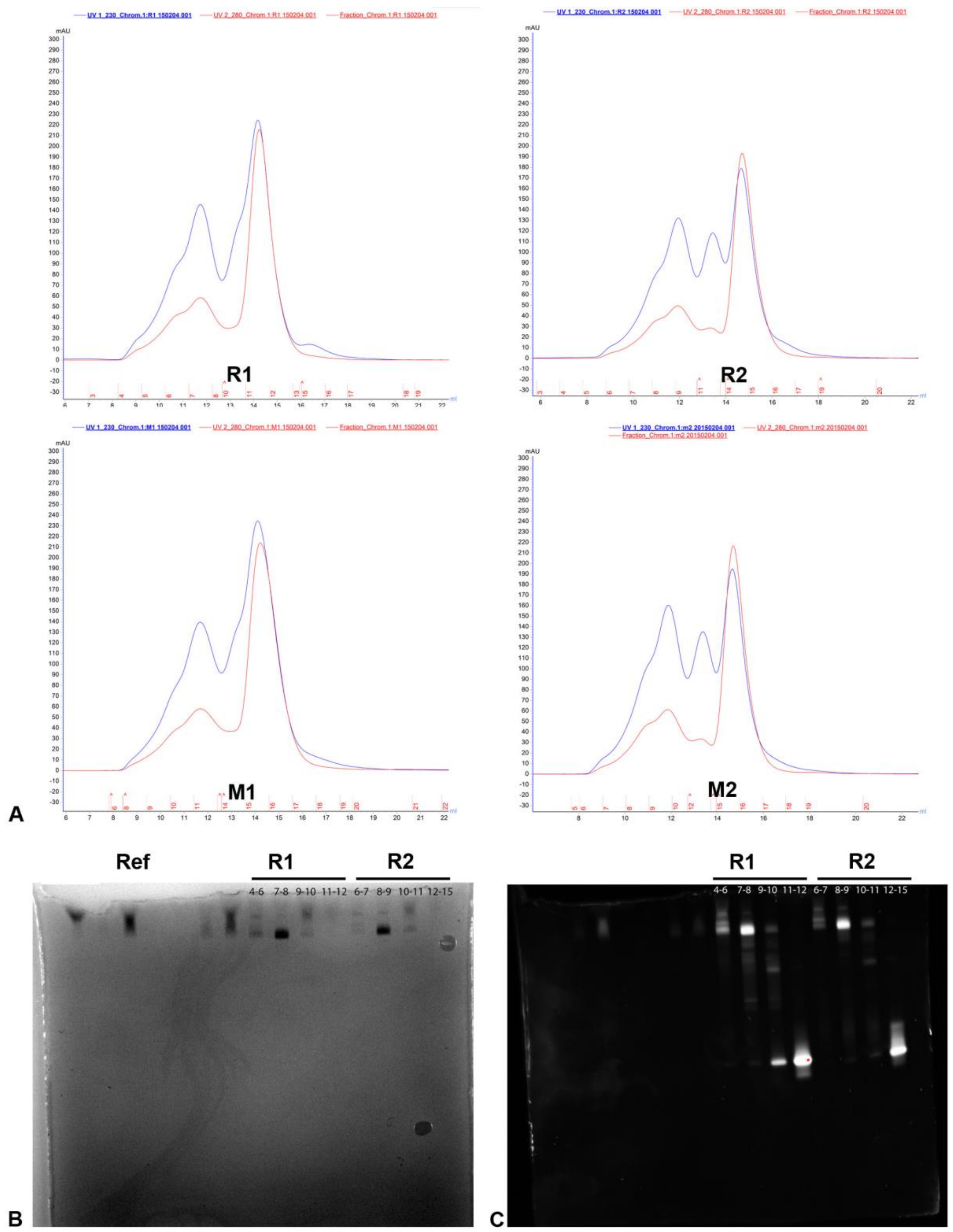

Supplementary Figure 4: Validation of conjugated proximity probes. HPLC purification and analysis (**a**) shows two peaks in absorption at 280 nm (red) and 230 nm (blue). The identities of the different fractions are confirmed by commassie protein staining (**b**) and Sybr safe DNA staining (**c**). Conjugates can easily be identified when comparing these images, in this conjugation fractions 7-8 was selected for R1 (donkey anti-rabbit antibodies conjugated to PH1 oligonucleotide) and fraction 8-9 for R2 (donkey anti-rabbit antibodies conjugated to PH2 oligonucleotide).

Supplementary table 1: Design 1. This design did not yield visible signal.

Activator	GCGACATAAGCAGAGACGGACTCGCAT
Proximity-hairpin 1	ATCGTCCTTCATCGGGCGACATAAGCAGCAGTGAATGCGAGTCCGTCTCTGCTTATGTCG CCCGATGAAGGACG-Biotin
Proximity-hairpin 2	GACCCTAAGCATACATCGTCCTTCATAAAAAGTATCTGCTTATGTCGCCCGATGAAGGAC GATGTATGCTTAGGG-Biotin
HCR hairpin 1	Fluorophore- ATGAAGGACGATGTATGCTTAGGGTCGACTTCCATAGACCCTAAGCATACAT
HCR hairpin 2	GACCCTAAGCATACATCGTCCTTCATATGTATGCTTAGGGTCTATGGAAGTC - Fluorophore

Supplementary table 2: Design 2 – reverse (not reverse complement) sequence of first design. This design yielded signal but signal generation was still relatively slow. Also a low amount of signal could be generated without the addition of Activator.

Activator	GACTCGCATTCACTGAATACAGCGGGCCTTCCTG
Proximity-hairpin 1	Biotin- GCAGGAAGGCCCGCTGTATTCAGTGAATGCGAGTCCAAGACGAATACAGCGGGCCTTCC TGCTACATACGA
Proximity-hairpin 2	GGGATTCGTATGTAGCAGGAAGGCCCGCTGTATTCGTCTAAAAATACTTCCTGCTACATA CGAATCCCAG
HCR hairpin 1	Fluorophore- TACATACGAATCCCAGATACCTTCAGCTGGGATTCGTATGTAGCAGGAAGTA
HCR hairpin 2	CTGAAGGTATCTGGGATTCGTATGTATACTTCCTGCTACATACGAATCCCAG- Fluorophore

Supplementary table 3: Design 3. No visible signal without addition of Activator. However initiator binds PH2 and can generate significant amounts of false positive signal (changed bases compared to Design 2 are marked in red; deleted bases are left as underscores).

Activator	GACTCGCATTCACTGAATACAGCGGGCCTTCATGTTACAGACGA
Proximity-hairpin 1	TCGTCTGTAACATGAAGGCCCGCTGTATTCAGTGAATGCGAGTC__AGACGAATACAGCGGGCCTTCATGTTACAGACGA
Proximity-hairpin 2	CTGGGAGTCGTCTGTAACATGAAGGCCCGCTGTATTCGTCT____TACTTCATGTTACAGACGACTCCCAG
HCR hairpin 1	Fluorophore-_ACAGACGACTCCCAG_TACCTTCAGCTGGGAGTCGTCTGTAACATGAAGTA
HCR hairpin 2	CTGAAGGTA_CTGGGAGTCGTCTGT_TACTTCATGTTACAGACGACTCCCAG-Fluorophore

Supplementary table 4: Design 4 (final). Missmatch between Activator and PH2 abolishes the unwanted interaction of those two oligonucleotides. Amplifier hairpins are optimized for higher speed. Last base of H2 has been changed from G to C so that previously occurring guanine quenching could be abolished (changed bases compared to Design 2 are marked in red). The underscored As in the Proximity Hairpins are spacer bases that were only used when the oligonucleotide was conjugated to an antibody.

Activator	GACTCGCATTCACTGAATACAGCGGGCCTTCATGCCACAGACGA
Proximity-hairpin 1	AAAAATCGTCTGTGGCATGAAGGCCCGCTGTATTCAGTGAATGCGAGTCAGACGAATACAGCGGGCCTTCATGCCACAGACGA
Proximity-hairpin 2	AAAAAGTGGGAGTCGTCTGTAACATGAAGGCCCGCTGTATTCGTCTTACTTCATGTTACAGACGACTCCCAC
HCR hairpin 1	Fluorophore-ACAGACGACTCCCACATTCTCCAGGTGGGAGTCGTCTGTAACATGAAGTA
HCR hairpin 2	CTGGAGAATGTGGGAGTCGTCTGTTACTTCATGTTACAGACGACTCCCAC -Fluorophore
